# Pituitary adenylyl cyclase-activating polypeptide modulates the stress response: the involvement of different brain areas and microglia

**DOI:** 10.3389/fpsyt.2024.1495598

**Published:** 2025-01-27

**Authors:** Anika Singh, Paul Shim, Sadaf Naeem, Shafiqur Rahman, Kabirullah Lutfy

**Affiliations:** ^1^ College of Pharmacy, The University of Rhode Island, Kingston, RI, United States; ^2^ Department of Biological Sciences, California State Polytechnic University, Pomona, CA, United States; ^3^ Institute of Pharmaceutical Sciences, Jinnah Sindh Medical University, Karachi, Pakistan; ^4^ Department of Pharmaceutical Sciences, College of Pharmacy, South Dakota State University, Brookings, SD, United States; ^5^ College of Pharmacy, Western University of Health Sciences, Pomona, CA, United States

**Keywords:** PACAP, stress, hypothalamus, pituitary, adrenal gland, BNST, amygdala, microglia

## Abstract

Stress is necessary for survival. However, chronic unnecessary stress exposure leads to cardiovascular, gastrointestinal and neuropsychiatric disorders. Thus, understanding the mechanisms involved in the initiation and maintenance of the stress response is essential since it may reveal the underpinning pathophysiology of these disorders and may aid in the development of medication to treat stress-mediated diseases. Pituitary adenylyl cyclase activating polypeptide (PACAP) and its receptors (PAC1, VPAC1 and VPAC2) are expressed in the hypothalamus and other brain areas as well as in the adrenal gland. Previous research has shown that this peptide/receptor system serves as a modulator of the stress response. In addition to modulating the stress response, this system may also be connected to its emerging role as neuroprotective against hypoxia, ischemia, and neurodegeneration. This article aims to review the literature regarding the role of PACAP and its receptors in the stress response, the involvement of different brain regions and microglia in PACAP-mediated modulation of the stress response, and the long-term adaptation to stress recognizable clinically as survival with resilience while manifested in anxiety, depression and other neurobehavioral disorders.

## Introduction

Stress is known as any threat caused by a stimulus that prompts a change in internal stability and elicits a biological response. Stress is recognized as the body’s internal response to the disruption of homeostasis that brings about physiological and behavioral changes. The stress response comprises of interconnections between the nervous, endocrine, and the immune systems which divert energy and resources to confront the stressor at hand. The body’s ability to regulate this response and restore homeostasis is coordinated primarily through the hypothalamic-pituitary-adrenal (HPA) axis. The HPA axis is the primary circuit which responds to homeostatic challenges with its final effector, glucocorticoids (GCs), that act upon central and peripheral targets (1). The HPA axis is a critical pathway in adapting to acute stressors; upon activation of HPA axis, stress responses are initiated by the release of corticotropic releasing hormone (CRH, also known as corticotropin releasing factor, CRF) and arginine vasopressin (AVP) from the hypothalamus that in turn activates the anterior pituitary to release adrenocorticotropic hormone (ACTH) hormone in systemic circulation. Elevated ACTH levels consecutively regulate GC synthesis especially cortisol (in humans) and corticosterone (in rodents). However, prolonged release of GCs can lead to inappropriate responses and psychopathologies (2). Thus, understanding the underpinning mechanisms modulating the stress response may represent potential target to regulate the stress response and may aid in the development of pharmacotherapy to treating stress-related disorders, such as posttraumatic stress disorder (PTSD), major depressive disorder (MDD), anxiety, and others.

## How is the stress response initiated and how is it turned off?

Activation of the HPA axis is initiated in the medial parvocellular subdivision of the PVN of the hypothalamus, which leads to the increased expression and release of CRH and AVP (3). Hypothalamic CRH travels through the hypophyseal portal circulation reaching the anterior pituitary and inducing ACTH release. Upon binding to CRH receptors in the anterior pituitary, CRH causes the release of ACTH, which travels through blood circulation and reaching the adrenal cortex, where it acts to stimulate the secretion of cortisol in humans and corticosterone in rodents (4). This coordinated central response providing quick responses to stress and promote HPA axis, metabolic, autonomic and hormonal signaling, leading to alterations in cellular excitability as well as synaptic and neuronal plasticity (5). Collectively, these peripheral-central effects mediate alterations in physiology and behavior that enable adaptation and survival. Eventually, the response is terminated through a negative feedback mechanism of GC acting at the pituitary and hypothalamic levels.

The limbic structures, and closely related regions, such as the amygdala, hippocampus, prefrontal cortex, bed nucleus of stria terminalis (BNST), and habenula, have been implicated in initiating and terminating the stress response with direct and indirect projections primarily to the levels of the hypothalamic PVN and anterior pituitary which are discussed below.

## Central regulation of stress

In the central nervous system (CNS), HPA axis, hippocampus, amygdala and the prefrontal cortex play important roles in stress regulation (6). Under chronic stress, regions such as hypothalamus and amygdala are crucial structures that integrate signals in the CNS and then propagate them to the periphery, largely via the autonomic nervous system and HPA axis (7). The amygdala, known for its role in emotion, anxiety, and fear memory acquisition, has accumulated interest in its involvement in the stress response, whereas the hippocampus supports determining the context in which such events take place (8). The amygdala, hippocampus and prefrontal cortex are linked with each other anatomically and functionally. For example, long-term potentiation in the hippocampus becomes reduced when there is a lesion in basolateral amygdaloid nucleus while stimulation of this nucleus facilitates the hippocampal long-term potentiation (9, 10). Additionally, lesions of the dorsal and ventral areas of the prefrontal cortex significantly hinder regulation of the stress response via circuitry within the hypothalamus (11). These findings point to the important interconnected role of these three major brain areas in stress regulation. The prominent collection of nuclei in amygdala includes the central amygdala (CeA), medial subdivision of the CeA (MeA), and the basolateral amygdala (BLA). The BLA is further divided into the lateral subdivision (LA) which receives afferent sensory inputs from the thalamus and cortex with efferent glutamatergic projections to the nuclei of the CeA (12, 13). Auditory fear conditioning paradigms in LA lesioned mice demonstrate reduced freezing behaviors compared to sham control where damage to both thalamo-amygdala and thalamo-cortico-amygdala pathways significantly reduced behavioral response to stressors (13, 14). Additionally, the amygdala has been shown to be an extrahypothalamic source of CRF and triggering sympathetic drive. Induced CRF overexpression in the CeA increased the CRF and AVP in the PVN with elevated plasma ACTH and corticosterone levels (15). This is further supported by lesions to the bilateral CeA of Rhesus monkeys that demonstrate blunted fear and anxiety-related behaviors with a significant decrease in cerebrospinal fluid CRF, plasma ACTH and cortisol levels (16). These findings suggest that the amygdala is a critical component of the central stress mechanism affecting hippocampal functioning.

Amygdala, hippocampus and prefrontal cortex are interconnected with each other (6) as well as with BNST, that is a central point in the stress regulation. The amygdala perceives a potential danger first; the hippocampus conceals environmental information associated with the stressors and the PFC modulates the emotional/stress response by assessing the information gathered from the amygdala and hippocampus (17). Previous research has shown that PFC exhibits reduced neuronal connections as a result of repeated stress, as well as simultaneous stress response impairs the executive functioning of PFC (18). Furthermore, PFC may influence the activity of the amygdala as amygdala and PFC have mutual anatomical connections. For example, lesions in the PFC decreases extinction of cued fear conditioning that is an amygdala-dependent task (19). Furthermore, Orem and colleagues (20) identified relationship between brain activity and the emotional response to stressors among 239 participants and concluded that the activity within the amygdala and PFC is important for the expression and regulation of behavioral and emotional changes observed in response to stress.

## Neuropeptides/neurotransmitters involved in the regulation of stress

Among the many pathways and mediators discussed above, neuropeptides and neurotransmitters are also important in the stress regulation. Neurotransmitters such as epinephrine, norepinephrine, dopamine, acetylcholine, glutamate and gamma aminobutyric acid (GABA) are also involved in the regulation of stress responses (21).

A number of neuropeptides, including vasopressin, neuropeptide Y, neuropeptide S, substance P, galanin, dynorphin, and pituitary adenylyl cyclase-activating polypeptide (PACAP), to name a few, have been associated with either the initiation or course of stress responses (22–24). Here, we review a neuropeptide PACAP and its neuroanatomical site of action in regulating the stress response.

## PACAP and PACAP receptors

Pituitary adenylyl cyclase-activating polypeptide (PACAP), a pleiotropic polypeptide widely distributed throughout the CNS (25–27) is found in two bioactive isoforms consisting of 38 and 27 residues (PACAP-38 and PACAP-27, respectively), with PACAP-38 as the predominant form (28, 29). PACAP-27 is a C-terminally truncated form derived from PACAP-38 after it was discovered that PACAP-38 contained an amidation cleavage site (30). The truncated PACAP-27 is 70% homologous to vasoactive intestinal peptide (VIP) and has a close resemblance to VIP precursor and gene construction (31). Additionally, the PACAP-38’s structure and its precursor are highly conserved within rats, sheep, and humans emphasizing the importance of this peptide across various vertebrate phyla (32).

PACAP acts as a neurotransmitter, neuromodulator, neurotrophic factor as well as serves as a neuroprotective factor against apoptosis. The peptide also plays a functional role in gut motility, circadian rhythm, and stress response (31). PACAP’s effects are mediated via PACAP receptors, PAC1, VPAC1 and VPAC2, which are G-protein coupled receptors (GPCRs). Due to the high homology between the amino acid sequences of PACAP and VIP, these peptides have comparable affinity toward VPAC1 and VPAC2 receptors. However, PACAP binds to another distinct receptor, PAC1 (PAC1R), with a higher affinity than VIP. PAC1 is a 495-amino acid protein with seven transmembrane domains (30) and is found in the CNS and peripheral nervous system. As stated above, PACAP by binding to these receptors regulates proliferation, differentiation, and cell survival during development as well as regulating the synthesis and release of neuroendocrine hormones (33). VPAC1 is composed of 457-amino acids and is primarily found in the cerebral cortex and the hippocampus, and VPAC2 comprises of 437 amino acids and is located in the central nucleus of the amygdala, hippocampus, thalamus, and hypothalamus. Activation of VPAC2 receptors leads to the stimulation of adenylyl cyclase activity (30).

PAC1 and VPAC1 receptors are involved in a variety of signaling cascades, including activation of adenylyl cyclase, protein kinase C (PKC), and calcium regulation. The enzyme adenylyl cyclase catabolizes adenosine triphosphate (ATP) to generate cyclic adenosine monophosphate (cAMP). This enzyme plays an important role in a variety of physiological responses. It regulates sugar and lipid metabolism, cell growth and differentiation, and olfaction. Research shows that the activation of PAC1R in tissue injuries caused a direct delay in apoptotic events thus improving cell survival. Furthermore, these results suggested that the alleviation of cellular damages from the increase in PAC1R signaling reduces cellular damage related to cerebrovascular trauma, neurodegeneration, and peripheral organ damages (34). Furthermore, the stimulation of PKC and calcium regulation are attributed to VPAC receptors via the phospholipase C (PLC) pathway through VPAC coupling to the Gq protein (35).

## PACAP as a regulator of the stress response

**Figure 1:**
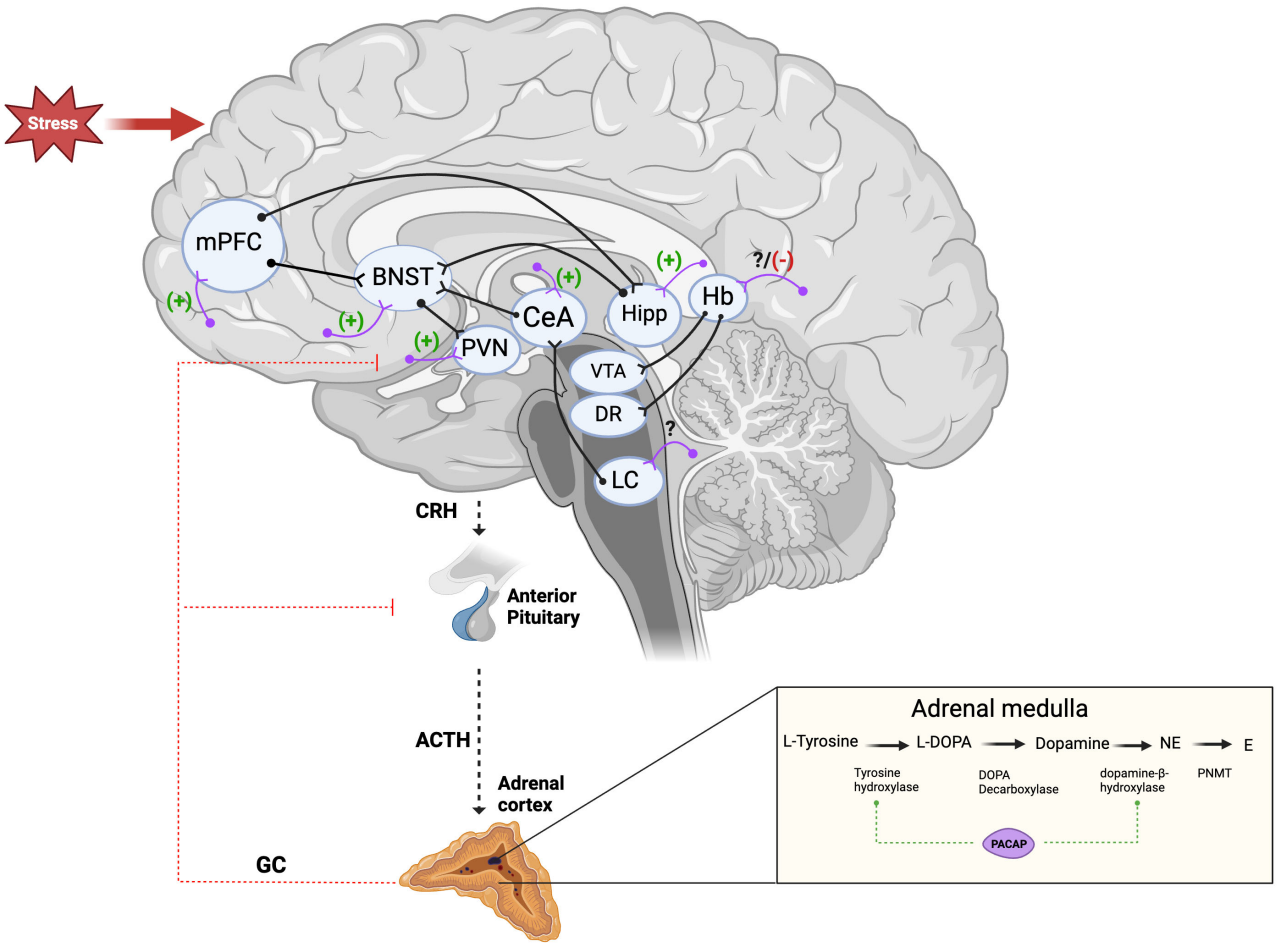
PACAP regulation of the stress response along the HPA axis and different brain regions. The black lines represent the projections and connections between the limbic structures. The BNST is shown as a major relay center with its numerous afferent and efferent connections. The purple lines represent PACAP neurons found within these regions. (+) and (-) represent if PACAP activates or inhibits the stress response in these regions, respectively. (?) unclear how PACAP regulates the stress response in this area. ACTH, Adrenocorticotropic Hormone; BNST, Bed Nucleus of Stria Terminalis; CeA, Central Nucleus of Amygdala; CRH, Corticotropin Releasing Hormone; Dorsal Raphe; E, Epinephrine; GC, Glucocorticoids; Hb, Habenula; Hipp, Hippocampus; LC, Locus Coeruleus; L-DOPA, L-dihydroxyphenylalanine; mPFC, medial prefrontal cortex; NE, Norepinephrine; PACAP, Pituitary Adenylyl Cyclase Activating Polypeptide; PVN, Paraventricular Nucleus of the Hypothalamus; VTA, Ventral Tegmental Area.

Evidence suggests that PACAP and its receptors play a variety of roles in the human body, more specifically this peptide/receptor system regulates the activity of the HPA axis and stress response due to its high abundance in the hypothalamus and other brain regions implicated in the stress response. It also regulates the adrenal medulla and controls the synthesis and release of epinephrine and norepinephrine in response to stress, preparing the subject for the “fight and flight” response (36). The PACAP and its receptors are present in different parts of the HPA axis and thus are well positioned to regulate the stress response by altering the level of CRH, ACTH, glucocorticoids, or epinephrine (**Figure 1**). Below we have provided some evidence for these regulatory actions of PACAP on the HPA axis and stress response.

## PACAP and CRH in regulating the stress response

The hypothalamic PVN has emerged as one of the most important stress control brain areas. Upon confronting the stress, CRH is released in the medial paracellular subdivision of the PVN (37). The release of CRH, a 41 amino acid peptide hormone, triggers the synthesis and secretion of ACTH (38). CRH-containing neurons are densely distributed throughout the PVN, BNST and are also expressed throughout the brain (39). A high level of PACAP and its receptor are co-localized with CRH neurons in the PVN (32, 40, 41). PACAP has been shown to increase CRH expression in the PVN (42, 43). Later studies present similar findings that PACAP alters CRF transcript through the cAMP/PKA pathway, as exogenous administration of PACAP produces elevated levels of phosphorylated cAMP response element-binding protein and an increase in Fos-immunoreactivity within CRH-containing neurons leading to significantly elevated corticosterone levels (44). Local PACAP administration into the PVN produces behavioral responses that closely mirror CRH activation in response to stressors and simulates corticosterone secretion (44).

Nr4a transcription factors in the hypothalamic PVN have been found to be closely coupled with HPA activation, leading to enhanced CRH mRNA expression (42, 43). Additionally, Stroth and colleagues demonstrate that upregulation of Nr4a transcription factors are PACAP-dependent as PACAP-deficient mice showed attenuated levels of transcription factors compared to wildtype mice. Studies involving a restraint stress in PACAP knockout mice show blunted levels of CRH transcripts and corticosterone compared to their wild-type counterparts (42, 43, 45). While PACAP increases CRH biosynthesis in the PVN, PACAP does not directly increase CRH secretion into the hypophysis (46). Recent evidence demonstrates a frontocortical descending PACAPergic input to the hypothalamus that regulates PVN CRH expression and is involved in the activation of the HPA axis (47). At the level of the PVN, PACAP plays a major role in prolonged HPA axis activation, where PAC1 appears to be the dominant receptor during sustained stress exposure, but other PACAP receptor types should also be examined in future studies (48). These findings suggest that PACAP within the hypothalamus acts to increase CRH transcripts.

PACAP-ergic nerve terminals have also been found to synapse on arginine-vasopressin (AVP) neurons in the supraoptic nucleus of the hypothalamus in rats (49). Studies suggest that PACAP may be acting through PKA-mediated phosphorylation and the opening of calcium channels inducing secretion of AVP (49). Further evidence in favor of this notion is that PACAP stimulates AVP and CRH transcription through the cyclic-adenosine monophosphate/protein kinase A (cAMP/PKA) pathway (50). Additionally, PACAP has been found to stimulate interleukin-6 gene transcription, a proinflammatory cytokine capable of regulating the HPA axis, and leading to elevated CRH and AVP promoter activity (50).

As stated above, local PACAP administration in the PVN closely resembles CRH in inducing behaviors associated with stressors. Evidence demonstrates that intra-PVN infusion of PACAP in mice displays similar phenotypic characteristics to CRH injections into the PVN, such as increased grooming and freezing behaviors (44, 51). Overall, these findings demonstrate that PACAP and its receptors are highly expressed in the PVN and other nodes of the HPA axis and this PACAP/receptor system stimulates the expression of CRF, leading to increased plasma corticosterone, and may be involved in stress-mediated neuropsychiatric disorders. Indeed, PACAP deficient mice have been shown to demonstrate reduced anxiety and depressive phenotypes during exposure to chronic and acute stressors (52, 53).

## PACAP and ACTH in regulating the stress response

Through interactions with its receptors on pituitary cells, PACAP increases the release of anterior pituitary hormones such as growth hormone, ACTH, prolactin, thyroid- stimulating hormone (TRH), and gonadotropins (54). In response to stressors, CRH as well as other peptides are released from the PVN and travel to the anterior pituitary to bind to cognate receptors on corticotropes, causing the release of ACTH. There is evidence that PACAP may regulate ACTH synthesis and release from the corticotrop cells. Chiodera and colleagues showed while infusion of a low dose PACAP did not alter circulating ACTH concentrations, the level of ACTH significantly increased with higher doses of PACAP (55). This finding suggests that systemic administration of PACAP dose-dependently stimulates ACTH secretions from the anterior lobe of the pituitary gland.

## PACAP and the adrenal cortex in regulating the stress response

The adrenal cortex is the outer region and the largest part of the adrenal gland. PACAP plays an important role in the regulation of adrenal cortical secretion and growth, mainly acting indirectly on its parenchymal cells. PACAP enhances ACTH secretion from pituitary corticotrop cells, and ACTH activates aldosterone and glucocorticoid production from adrenal zona glomerulosa and zonae fasciculata-reticularis, respectively, as well as stimulates adrenal hypertrophy (56, 57). VIP and PACAP enhance pituitary ACTH secretion, either directly or via the release of CRH, and thus may increase mineralo- and glucocorticoid secretion from the adrenal cortex in response to stress (58). Within the adrenal gland, the highest density of PACAP and PAC1 receptors are found in the adrenal medulla (53, 59, 60). Studies have found that restraint stress-induced secretion of corticosterone is attenuated in mice lacking PACAP compared to their wildtype controls, further proving the importance of PACAP in the stress response (61). Furthermore Lehmann et al. (53) discovered lipopolysaccharide administration in PACAP deficient mice showed attenuated corticosterone secretion and animals failed to develop stress behavior after exposure of stressors. These results suggest that PACAP is more prominently involved in emotional stress-dependent corticosterone secretion. Research also established c-Fos expression increased in the CRH but not AVP neurons in the PVN or medial subdivision of the CeA (MeA). These responses were significantly decreased in PACAP-deficient mice, providing further support for the involvement of PACAP in restraint stress-induced corticosterone secretion (61). Research was also conducted to measure corticosterone levels in mice lacking the PACAP gene, following an injection of trimethyltin (TMT). It showed that TMT caused a dramatic increase in plasma corticosterone levels in wild-type mice, while there were no significant changes in corticosterone secretion in PACAP-deficient mice. Overall, these findings suggest that PACAP plays a functional role in the stress response and corticosterone secretion, but the type of stressors may be important.

## PACAP and the adrenal medulla in regulating the stress response

Alongside the HPA axis, the hypothalamo-sympathoadrenal (HSA) system, which includes the adrenomedullary splanchnic nerve, is another closely related neuroendocrine regulator of homeostatic challenges. Briefly, presynaptic splanchnic nerve terminals that synapse on postsynaptic cholinergic/nicotinic receptors on chromaffin cells cause depolarization of these cells and opening of voltage gated calcium channels, allowing the entry of calcium in these cells and leading to exocytosis of large dense core vesicles containing catecholamines [reviewed in (62, 63)]. Binding of acetylcholine (ACh) to nicotinic acetylcholine receptors (nAChRs) is known to be the primary mechanism of medullary secretion, but this effect last for a short time due to the desensitization of nAChRs (64). Thus, a prolonged secretion of catecholamines may require a mechanism other than nAChR activation by ACh. PACAP receptors present adjacently with the vesicular ACh transporter in the adrenal presynaptic nerve terminals and by acting as catecholamine secretagogues represent a potential mechanism for prolonged catecholamine secretions in response to stressors (36, 65, 66). Recent evidence suggests that PACAP and ACh stimulates secretary pathways via independent mechanisms, in which PACAP’s actions involve exchange proteins directly activated by cAMP and PLCε (67). PACAP activates the adrenal gland through the cAMP/PKA pathway by gene regulation of tyrosine hydroxylase (TH) and dopamine beta-hydroxylase (DBH), two catecholamine synthesizing enzymes (68–70). This finding correlates with the finding of Stroth and colleagues who reported that PACAP knockout animals showed attenuated catecholamine synthesis by direct high-frequency stimulation of the splanchnic nerve in native adrenal slices while wildtype mice showed increased depolarization of adrenal medulla catecholamine transcripts (71). As stated above, PACAP and its receptor have been identified to colocalize with ACh on splanchnic nerve terminals and in the adrenal medulla, where they upregulate TH and phenethanolamine N-methyltransferase (PNMT) expression (70). The latter enzyme is responsible for the conversion of norepinephrine to epinephrine in the adrenal medulla and some central adrenergic neurons. Evidence suggests that acute stress-induced prolonged secretion of catecholamines is mediated by PACAP and its receptors that in turn promote a concordant rise in serum glucose levels (59, 72). PACAP-deficient mice exposed to metabolic stressors (i.e., prolonged hypoglycemia) displayed significantly lower glucose levels and were unable to survive following exposure to such stressors (59).

## PACAP and medial prefrontal cortex and regulation of the stress response

The prefrontal cortex has been shown to provide an inhibitory input to the hypothalamus, thereby regulating the HPA axis and thus the stress response. In addition, the medial prefrontal cortex (mPFC) is known for its role in executive function and affective information processing (73). A bulk of evidence points to mPFC having an inhibitory effect on the HPA axis through indirect synapses mediated by structures such as the bed nucleus of stria terminalis (BNST). mPFC is most sensitive brain area to the detrimental effects of stress. Even a mild acute but uncontrollable stressor can cause a rapid and dramatic loss of prefrontal cognitive abilities, and more prolonged stress exposure causes architectural changes in prefrontal dendrites (74). Martelle et al. (75) explored the effect of PACAP on the stress response by microinjecting PACAP (1 ug) in the PFC and after five days passive avoidance paradigm testing revealed that PACAP infused mice displayed increased latency and memory retention in crossover consistent with anxiety-like behaviors (75). Two weeks later a significant rise of PACAP expression was seen in putative pyramidal neurons. Thus, the authors concluded PACAP plays a functional role in stress responses and in processing of fear memories (75). It suggests that PACAP is involved in prefrontal processing of traumatic stress and fear learning. Preclinical research suggested that PACAP receptors are confined to the PFC, which regulates fear memories by interconnecting with hippocampus and BNST (76). Recent evidence shows that 2-h restraint stress causes an increase in c-fos expression and CRH mRNA levels, a response blunted in the absence of PACAP in the mPFC neurons projecting to the hypothalamus (47).

PACAP and its receptor have been localized in the infralimbic cortex (IL), where PAC1 is found on GABA neurons and interneurons, while PACAP on glutamatergic cells. Additionally, PACAP infusions to the IL induce sympathetic activation which confirms known functions within this region. Increased expression of PAC1 transcripts in the prelimbic cortex (PL) have been reported in female rats in response to cued fear conditioning suggesting females may be more responsive to PACAP signaling during affective memory processing (77). In human studies, sex-related differences in plasma PACAP levels have been noted in participants with PTSD-like symptoms. This difference has been suggested to be influenced by estrogen response elements on the expression of PAC1 receptor gene. A recent study discovered a sexual dimorphism of PACAP expression in the paraventricular nucleus of the thalamus (PVT), a region involved in affective and motivated behaviors, to be significantly increased in females compared to male mice (78). Studies using VPAC2 knockout mice suggest that this receptor type is most likely involved in fear extinction and retrieval processes (79). Consequently, VPAC2R has been reported to play an important role in the dendritic organization of the PL and IL (80). In PACAP knockout mice, FosB immunostaining was elevated in MPFC after exposure to chronic stress compared to wildtype mice, showing that endogenous PACAP is normally required to modulate stress-responsive circuit during chronic stress (53).

## PACAP and hippocampus in regulating the stress response

The hippocampus, located in the medial temporal lobe of the brain, is involved in memory formation, such as contextual learning, and regulation of behavior. Upon stimulation of stress response and HPA axis, hypothalamus released corticotrophin releasing factor (CRF) that in turn stimulates the release of ACTH from anterior pituitary, that is further responsible for the release of GCs from adrenal cortex. This stimulation of HPA axis is counter-regulated by negative feedback mechanism (81). The hippocampus has been implicated to have an inhibitory influence over the HPA axis with evidence pointing to the ventral subiculum (82–84). HPA axis is controlled by neuroendocrine neurons located in the medial PVN of the hypothalamus. PVN is one of the primary sites of glucocorticoid negative feedback regulation of the HPA axis. Anatomical studies indicate that direct innervations to the PVN have yet to be identified, but subicular projections to the BNST have been identified which further implicate the BNST as a major relay center that receives abundant input from the limbic regions and hippocampus sends outputs to PVN via the BNST (85).

The ventral hippocampus contains a high density of glucocorticoid (GC) receptors that is why stress can so readily impede this part of the brain. Evidence suggests that GC receptors in the hippocampus affect the negative feedback termination of the stress response. This action occurs through the stimulation of hippocampal neurons which ultimately leads to a decrease in neuronal activity in the parvocellular division of the PVN, thus hindering GC secretion (3). In animal models, GCs hypersecretion leads to reduced number of GC receptors in the hippocampus (86, 87). PACAP receptors, such as PAC1Rs, have been found in hippocampal dentate hilar mossy cells, and PVN, studies revealed that potentiation of DG synapses is impaired in PAC1 knockout mice (88). The hippocampus seems to play a pivotal role in the generation of long-term memory through NMDA and AMPA receptors. It is evident that PACAP enhances both NMDA and AMPA currents in the hippocampus through PAC1R, activation of which in CA1 hippocampal neurons increases evoked NMDA currents via the cyclic AMP/PKA pathway that modulates LTP (89–91). Evidence also shows a dose-dependent action of PACAP on AMPA-receptor mediated glutamatergic transmission at the CA3-CA1 synapse (92).

In DG cells, PACAP has been reported to enhance action potential excitability through mitogen-activated kinases/extracellular signal-regulated kinase signaling (93, 94). PAC1 has been identified to be more prominent than the other PACAP receptor types in the hippocampus. Immunohistochemical studies of the hippocampus describe PAC1 as being localized to presynaptic mossy fiber terminals and also on CA3-CA1 pyramidal cells (76, 95, 96). VPAC1 and VPAC2 have been identified to be dispersed throughout all regions of the hippocampus (95). Local application of PACAP38 in the CA1 region was found to enhance field excitatory postsynaptic potentials responses and alter synaptic strength (97). This is further supported in the CA1 pyramidal neurons showing increasing excitability in the presence of PACAP38 and PACAP27 (98). These studies suggest that PACAP may modulate the stress response or associated behavioral changes via an action within these hippocampal regions.

Glutamate NMDA receptors are associated with learning and memory processes such as contextual fear conditioning and long-term potentiation. PACAP has been shown to modulate the NMDA receptor-mediated activity and learning processes (99). PACAP38 has been found to enhance NMDA-receptor current in the CA1 region primarily through the Gq pathway (90). Studies in knockout mice show that PAC1R is involved in modifying patterns of associative learning, contextual fear conditioning, and synaptic plasticity (88).

## PACAP and locus coeruleus in stress response

LC is characterized by its role in arousal and response to stressful stimuli. LC is a major source of adrenergic neurons in the brain with connections to the cortex and hippocampus (100). Adrenergic-containing fibers from the LC have been identified to project to the basolateral amygdala promoting anxiogenic behaviors (101). Additionally, elevated c-Fos expression has been noted in the CeA in stressed rats, and this neuronal communication between LC and CeA has been shown to produce acute anxiety-like behaviors (102). CRH-containing neurons have also been reported as a key modulator of LC activity. Known extrahypothalamic sources of CRH, originating from the CeA and mPFC, have been identified to project to the LC (102, 103). Application of CRF on LC neurons demonstrates increased excitability and elevated levels of plasma norepinephrine (104, 105). While CRF has been known to increase tonic firing of LC neurons, the release of endogenous opioids in the LC has been shown to counteract the effects of CRF, returning activity to baseline levels (106). Evidence shows that the LC contains close interactions with both the norepinephrine and corticotropin systems suggesting that it is another key regulator of the sympathetic response (107).

It has been known that a high level of PAC1 receptor populates in the LC (41), but studies involving PACAP’s role in this brain region are limited. The high density of this receptor type in the LC has produced an interest in symptoms of morphine withdrawal. PAC1 knockout mice interestingly display increased withdrawal phenotypes (108). Martin and colleagues expected PAC1 activity to increase withdrawal symptoms as PKA-dependent CREB phosphorylation is known to be elevated during withdrawal; however, the LC may not be solely responsible for withdrawal symptoms and this process may be mediated by a different pathway. Additionally, LC-PAC1 has also been indicated in sex-dependent alteration of energy metabolism, but not fear expression in mice exposed to stress (102, 109). Future studies should focus on PACAP’s effect on the LC-NE system in the context of HPA response.

## PACAP and habenula in stress response

Habenula plays a critical role in motivation and emotional regulation as well as in stress-induced psychopathologies. Hb can be divided into two distinct nuclei: the medial (MHb) and lateral (LHb) habenula (110). The LHb is a relay center that receives inputs from various limbic structures, such as the mPFC, BNST, PVN, and lateral preoptic area [reviewed in (111)]. The LHb contains glutamatergic output projections to both 5-HT and dopamine circuits such as the dorsal raphe and the ventral tegmental area (VTA), respectively (111). GABAergic interneurons have also been identified on the LHb regulating output from this region (112, 113). The LHb has been indicated to play a role in contextual learning, depression, stress processing, motivation and avoidance learning (112–114). Exposure to stress has been shown to produce anhedonic behaviors in animals due to altered reward responses indicating that stress causes a negative shift in LHb signaling of reward and its omission (115). The mechanism behind the anhedonic behaviors after stress exposure may be explained by an altered reward signaling pathway as stress changed the polarity of neuronal responses to rewards, making them appear as punishment signals, in this way aberrant LHb signaling during stress leading to motivational impairment (116). LHb is a hub that convert negative affective states information and dysregulates the reward system. Upon stress exposure glutamatergic synaptic transmission is enhanced in LHb, as NMDA-dependent neuronal firing is observed in LHb neurons of mice that generated negative aversive states (117). Neuroanatomical and morphological alterations have been noted in rats, as chronic stress exposure leads to a reduction in cell volume and number of neurons in both the LHb and MHb (118). Furthermore, long term potentiation in subregions of the LHb has been shown to occur during exposure to acute stressors (119). CRF within the LHb has been shown to increase neuronal firing rate of glutamate and excitability through reducing GABAergic transmission from presynaptic terminals (120). CRF has been reported to increase glutamate activity and excitatory postsynaptic currents within the LHb in rats with a history of chronic alcohol use (121).

PACAP-expressing neurons have been found in the LHb (76), but PACAP’s role in this region has not been extensively explored. PACAPergic and glutamatergic neurons have been identified to be co-expressed within the LHb and MHb subdivisions (122). PACAPergic neurons of the LHb have been found to project on the raphe nuclei, rostromedial tegmental nucleus, and less densely to the VTA (110). ICV PACAP infusions in rats showed a decrease in c-Fos expression in the LHb, and resulted in a reduction in freezing behaviors (123). Levinstein and colleagues demonstrated that activation of PACAPergic neurons in the LHb produced anxiolytic behaviors and increased locomotion which is contrary to the known role of the LHb as a whole This may suggest that LHb PACAP neurons are producing differential behavioral phenotypes than LHb activation as a whole during stress exposures (110). It seems that LHb-PACAPergic neuronal activation during the stress response diminishes the negative consequences of aversion and reduces fear and anxiety-associated behaviors (124).

## PACAP and amygdala in stress response

Amygdala is known for its role in emotion, anxiety, and fear memory. It detects stress and informs the HPA axis to respond (125). The CeA acting as a hub for negative emotional processing and interconnected with GABA and glutamate projection neurons. A prominent collection of nuclei in this region includes the CeA, medial subdivision of the CeA (MeA), and the BLA. CeA receives excitatory glutamatergic projections from BLA and sends GABAergic afferent signals to CeM. The CeM serves as hub of the output nucleus which sends GABAergic signals to downstream efferent regions that control stress and fear responses (125). Amygdala is known to mediate the stress response through gaining information from sensory modalities (126). The stimulation of the amygdala afferents to the hypothalamus can activate the HPA axis, leading to the release of glucocorticoid into the systemic circulation (3). Research has shown that CRF expression in amygdala can mediate the adequate behavioral responses to stress and increase the anxiety-like behaviors (127).

CeA serves as a center for negative emotional processing and contains GABA interneurons and GABAergic projections that regulate stress and fear responses and plays an important role in brain’s inhibitory circuit (125, 128). In contrast, BLA contains both glutamatergic and GABAergic interneurons that participates simultaneously in somatic and behavioral responses to stressful stimuli (125).

The CeA is thought to act through a GABA-GABA disinhibitory mechanism by involving structures such as the BNST, containing abundant neurons projecting to the PVN (129). Additionally, the amygdala has been shown to be an extrahypothalamic source of CRF and trigger the sympathetic drive. *In vitro* studies have shown CRF overexpression after chronic stress exposure in the CeA, led to increased CRF and AVP in the PVN along with elevated plasma ACTH and corticosterone levels (15). This is further supported by results from bilateral lesions of the CeA in Rhesus monkeys that demonstrated blunted fear and anxiety-related behaviors with significant decreases in cerebrospinal fluid CRF, and plasma ACTH and cortisol levels (16).

PACAPergic neurons are abundantly present in CeA from the lateral parabrachial nucleus and dorsal vagal complex of the brainstem (130), and PAC1 is expressed throughout the CeA. Iemolo et al. (131) showed that intra-CeA PACAP-38 infusions directly accelerate the HPA axis and increases their anxiety-like response towards stressors. Indeed, Sieglie and colleagues reported that 10 days of chronic social defeat stress (CSDS) causes a significant increase in PACAP levels selectively in the CeA of PAC1R knockdown mice and prevents the chronic social defeat stress-induced increase in anxiety-like behavior (132). These findings suggest that PACAP is needed to change the activity of the stress circuit during chronic social defeat stress and regulate the altered mood and motivation following stress exposure. In addition, the CeA PACAPergic system helps to regulate anxiogenic and stress coping behaviors via PAC1 to restore normal function of CeA to maintain GABA release in the CeM. These authors also have shown that activation of lateral parabrachial PACAPergic neurons to the CeA induces anxiety-like behaviors (133). Varodayan and colleagues studied PACAPergic regulation in the CeA during restraint stress in male Wistar rats. They subjected the animals to either restraint stress or control conditions, and alterations in PAC1 receptor level and cellular functional changes by PACAP were assessed using immunohistochemistry and electrophysiology, respectively. They found that exogenous PACAP38 increased GABA release through the presynaptic PAC1 receptor. This response was attenuated in animals exposed to restraint stress. Tangentially, they found that single restraint stress decreases PAC1 immunoreactivity in the CeM. Overall, the results suggested that PACAP/PAC1 receptor signaling in the CeA is complex and diverse. The stress mediated GABA release in CeM is regulated by PACAP expression. On the other than hand, a single restraint stress reduces the impact of PACAP/PAC1 on the CeM GABA system (125).

The BLA PACAPergic projections to the intercalated cells microcircuit has also been implicated in contextual fear (134). Higher circulating PACAP along with greater amygdala connectivity with posterior cingulate cortex and left angular gyrus in women but not men with PTSD (135). Likewise, a single nucleotide polymorphism in the PACAP receptor gene has been reported in patients with PTSD which may contribute to a dysregulated fear circuitry (136).

## PACAP and BNST in regulating the stress response

Endogenous PACAP in the BNST is known to mediate stress responses and induces anxiety-like behaviors in rodents. In addition, mice that were treated with PACAP exhibited stronger anxiety-like behaviors with increases in weight loss. Roman et al. (137) reported direct PACAP administration in the BNST decreased entries in elevated plus maze, open field and novel object avoidances (137). Additionally, chronic stress exposure increased PACAP levels primarily in the dorsolateral BNST and caused a slight increase in the PAC1 receptor expression (138). Chronic variable stress was found to be anxiogenic and so was local PACAP administration in the BNST. PACAP was found to increase startle amplitude at 0.5µg dose and more robustly at 1.0 µg. This result also proved that chronic stress increases BNST neuroplasticity and heightens anxiety-like behaviors by a PACAP mediated mechanism (138). PACAP-deficient mice exposed to forced swim test exhibited reduced c-fos expression in different BNST nuclei as well as in the ventral septum and dorsal raphe nucleus (139). Immunohistochemistry showed a dense network of PACAPergic fibers in the lateral part of the BNST. VIP-immunoreactivity was also found in the lateral part of BNST but covers a larger area than PACAP. However, PACAP-immunoreactivity is not limited to this area but was also seen in the medial part of BNST (140). Thus, PACAP has the ability to affect CRH levels through PACAP/VIP receptor subtypes in these regions. In a recent study, PACAP levels were found to be higher in the BNST of submissive mice, implicating PACAP in the adaptation of dominance hierarchies (141). In another study, BNST PACAP expression was increased in the oval nucleus of BNST after chronic stress (137). However, additional studies are necessary to identify the origins of the PACAPergic fibers and VIP in the dorsal subdivision of the lateral part of the BNST.

Exogenous PACAP in the BNST shows many different responses. It was found that the effect of PACAP injected locally in the BNST of male and female rats resulted in increased secretion of corticosterone compared to the saline-treated control group even though this was only induced with injections of 1.0 micrograms of PACAP. However, PACAP injection in the lateral ventricle just above the BNST did not increase corticosterone secretion showing the site-specific effect of PACAP in increasing the stress response (142). Further studies have examined what behavioral and endocrine changes would occur after a BNST PACAP infusion in adult male and female Sprague Dawley rats exposed to chronic variate stress. The results showed that only the stressed males showed an increase in corticosterone levels after 30 minutes as well as an increased startle amplitude, where the same stressor did not change the startle response in female rats (37). In addition, PACAP injections in the PVN, in animals exposed to mild stress, body grooming and face washing were observed with decreases in rearing and locomotor activity both at 10 minutes and 2 minutes post PACAP injection. It is possible that the lack of locomotor activity could be compensated by the larger increase in body grooming (51). This proves that preceding stressors did in fact sensitize the behavioral and endocrine responses to PACAP injected into the BNST in male rats and PACAP injection in the PVN imitates stress. These actions of PACAP were not mimicked by VIP, suggesting that PACAP is acting via BNST PAC1 receptors (37, 143). Finally, research has been conducted to analyze the effect of PACAP administered in the CeA and BNST on startle amplitudes in adult male Wistar rats using the acoustic startle response. Results showed increases of startle amplitude following injection of PACAP, but not VIP, in the BNST and CeA. Foot shock stress increased startle amplitude and PACAP expression in the BNST and CeA. The effect of stress was blocked by a PACAP antagonist, PACAP 6-38, suggesting that stress increases startle amplitude by the PACAPergic system. However, studies are required to assess whether this action of PACAP is exerted via the extrahypothalamic region or HPA axis. Thus, further studies might require injecting a CRH antagonist in the hypothalamus and to assess whether the action of BNST PACAP can be mediated by CRH release in the hypothalamus (144).

## PACAP, stress and microglia

Microglia, as resident macrophages in the CNS, execute essential functions to maintain brain homeostasis and neuronal survival (145). They respond to noxious stimuli by releasing inflammatory cytokines such as interleukin (IL)-1β, IL-6, tumor necrosis factor-α (TNF-α) and chemokines (146). Emerging evidence indicates that activation of microglia, is critically involved in mediating inflammatory responses to various stresses that can serve as a major trigger for numerous stress-related neuropsychiatric disorders, such as Parkinson’s, Alzheimer’s, Huntington’s and depression (147). Furthermore, previous studies have shown that stress led to microglial activation, changed morphology, increased phagocytic capacity, and altered transcriptional profile that result in premature, progressive and chronic neural cell loss (148). The neuropeptide PACAP exert neuroprotective and immunomodulatory activities throughout the central and peripheral nervous systems. Activation of this endogenous neuropeptide may interfere with stress processes to promote glial cell survival and myelin self-repair (149). The neuroprotective actions of PACAP have been identified extensively in several research papers for neurodegenerative diseases (149–153). Intraperitoneal injection of PACAP with graded doses in experimental autoimmune encephalomyelitis mice model revealed that PACAP suppress pro-inflammatory cytokines induction through microglia (154). Similarly, in MPTP (1-mthyl-4-phenyl-1,2,3,6-tertrahydropyridine) induced Parkinson’s model, PACAP administration protected dopaminergic neuronal degeneration (155). Furthermore Broome et al. (150) reported cotreatment with PACAP or VIP prevented rotenone-induced increase of nitrous oxide, matrix metalloproteinase (MMP)-9 and IL-6 that plays a critical role in dopaminergic neuronal loss. Another study showed that long-term PACAP administration in Alzheimer’s (AD) transgenic mice improved cognitive function (156). Preclinical and clinical evidence suggests that multiple exposures to stress disrupt the homeostasis between inflammatory response and neuroprotective mechanisms, and lead to neuronal loss and cognitive decline (157–159). Cognitive decline has been associated with a decrease in brain-derived neurotrophic factor (BDNF) levels. Zink and colleagues (160) reported decreased expression of BDNF in hippocampal CA3 and dentate gyrus of PAC1-receptor-deficient mice. Several AD transgenic mouse models and human AD temporal cortex have showed down-regulation of PACAP receptors (161). The rescue of memory decline is linked with an increase of BDNF levels. PACAP is suggested to prevent cognitive decline via promoting protein expression of BDNF that participates in neuronal plasticity and essential for learning and memory (162).

**Figure 2:**
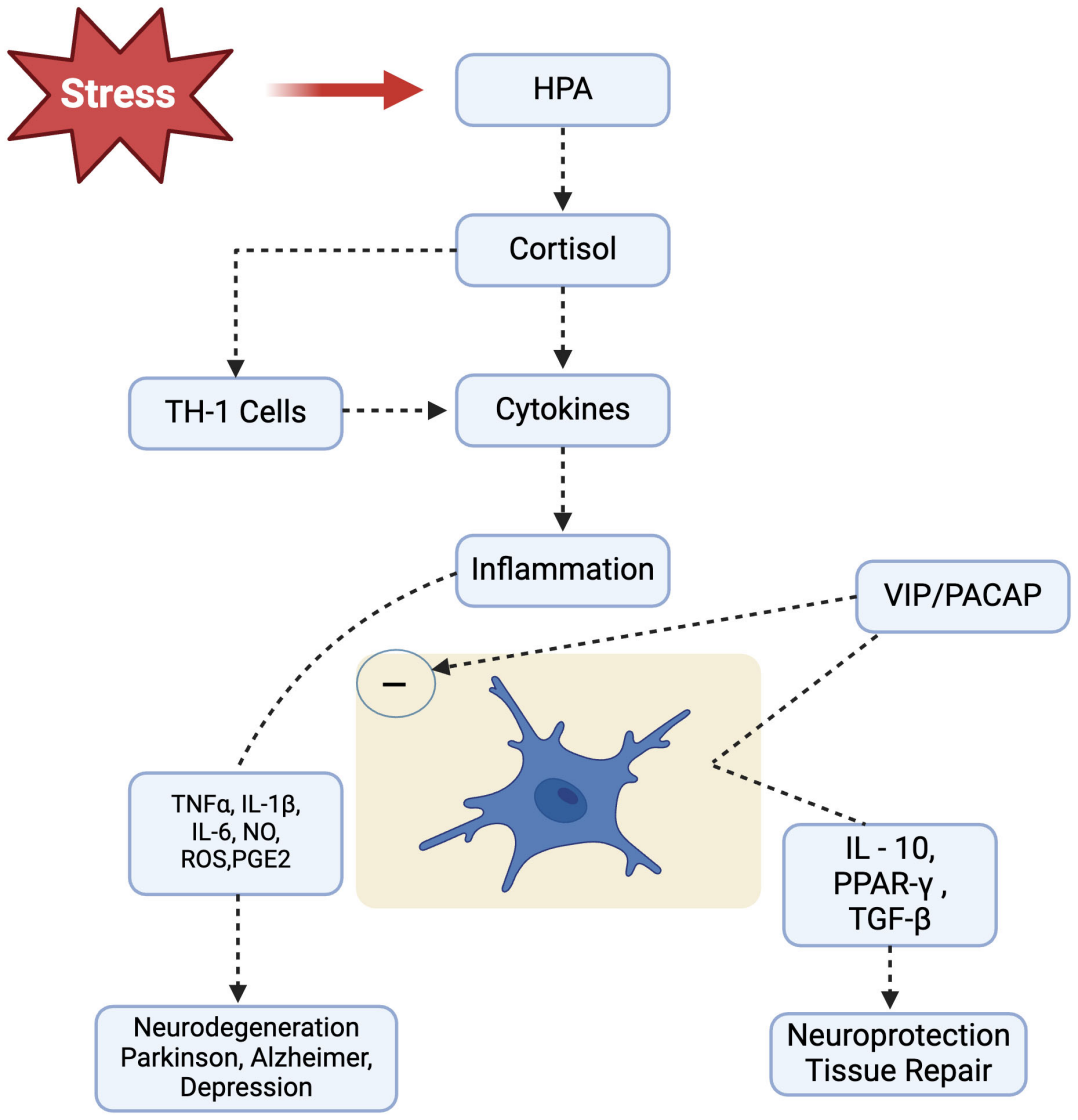
Stress leads to HPA axis activation and causes consistently increased cortisol levels in our body. Following cortisol release, Th1-mediated cellular immune response is initiated that produces plethora of inflammatory mediators that promote and perpetuate the inflammatory response, potentially leading to neurodegenerative diseases, such as Parkinsonism, Alzheimer’s and Depression). Neuropeptides VIP/PACAP, acting through specific receptors present on microglial cells, are able to modulate microglial response and inhibit the release of inflammatory mediators while favoring development of an alternative activation program. HPA, Hypothalamic Pituitary Adrenal; IL-1β, Interleukin-1beta; IL-6, Interleukin-6; IL-10, Interleukin-10; NO, Nitric Oxide; ROS, Reactive Oxygen Species; PACAP, Pituitary Adenylyl Cyclase Activating Polypeptide; PGE2, Prostaglandin E2; PPARγ, Peroxisome Proliferator-Activated Receptor-gamma; TGFβ, Transforming Growth Factor-beta; TNFα, Tumor Necrosis Factor-alpha.

Moreover, it has been suggested that PACAP and VIP might be exerting their neuroprotective effects in part by acting directly on microglial cells as microglia-deactivating factors (**Figure 2**). Expression of VPAC1 and PAC1 was detected in rat microglia, after LPS administration in rat’s spinal cord transaction, where these receptors VIP and PACAP significantly suppressed LPS induced TNF-α (163, 164). These neuropeptides signaling systems might be involved in attempts to minimize the impact of neuronal injury and cure neurodegenerative disorders.

## Concluding remarks

PACAP is involved in the regulation of many physiological responses. This review summarized the current literature regarding the influence of PACAP on the stress response, involving the HPA axis as well as extrahypothalamic brain regions. PACAP acts at different levels along the HPA axis to alter the stress response. PACAP acts at the level of the PVN to alter the expression of CRH. It also serves to cause the secretion of ACTH from the anterior pituitary. At the level of the adrenal gland, it may cause the secretion of glucocorticoids and alter the expression of catecholamine biosynthetic enzymes, TH, DBH, and PNMT. All these actions could promote the stress response. However, further studies are needed to determine whether PACAP is released in these brain regions. Although the HPA function are important to determine the susceptibility of the body to stress or insults during the life process, this is also the main clinical correlates of stress-related neurodegenerative disorders that represent a significant public health concern. In this context, PACAP/VIP system exert anti-inflammatory and neuroprotective effect through their receptors PAC1, VPAC1, and VPAC2. Given the diverse signaling of PACAP in the CNS, PACAP analogs/agonists can be considered as therapeutic option for stress related neuropsychiatric disorders. Future studies are required to design potent PACAP targeted medications that would be a promising therapy for neuropsychiatric and neurodegenerative disorders.
